# Constant ratio between the genomic components of bipartite begomoviruses during infection and transmission

**DOI:** 10.1186/s12985-023-02148-2

**Published:** 2023-08-21

**Authors:** Yu-Xin Xiao, Di Li, Yi-Jie Wu, Shu-Sheng Liu, Li-Long Pan

**Affiliations:** 1 Ministry of Agriculture Key Lab of Molecular Biology of Crop Pathogens and Insects, Key Laboratory of Biology of Crop Pathogens and Insects of Zhejiang Province, Institute of Insect Sciences, Zhejiang University, 310058 Hangzhou, China; 2 The Rural Development Academy, Zhejiang University, 310058 Hangzhou, China

**Keywords:** Bipartite begomovirus, Ratio between DNA-B and DNA-A, Infection, Transmission, Squash leaf curl China virus, Sri Lankan cassava mosaic virus

## Abstract

The genomic components of multipartite viruses are encapsidated in separate virus particles, and the frequencies of genomic components represent one of the key genetic features. Many begomoviruses of economic significance are bipartite, and the details of the association between their genomic components remain largely unexplored. We first analyzed the temporal dynamics of the quantities of DNA-A and DNA-B and the B/A ratio of the squash leaf curl China virus (SLCCNV) in plants and found that while the quantities of DNA-A and DNA-B varied significantly during infection, the B/A ratio remained constant. We then found that changes in the B/A ratio in agrobacteria inoculum may significantly alter the B/A ratio in plants at 6 days post inoculation, but the differences disappeared shortly thereafter. We next showed that while the quantities of DNA-A and DNA-B among plants infected by agrobacteria, sap transmission and whitefly-mediated transmission differed significantly, the B/A ratios were similar. Further analysis of gene expression revealed that the ratio of the expression of genes encoded by DNA-A and DNA-B varied significantly during infection. Finally, we monitored the temporal dynamics of the quantities of DNA-A and DNA-B and the B/A ratio of another bipartite begomovirus, and a constant B/A ratio was similarly observed. Our findings highlight the maintenance of a constant ratio between the two genomic components of bipartite begomoviruses during infection and transmission, and provide new insights into the biology of begomoviruses.

**Supplementary Information:**

The online version contains supplementary material available at 10.1186/s12985-023-02148-2.

## Introduction

Viruses pose considerable threats to human health and crop production worldwide [1, 2]. As a group of obligate parasites, most viruses are characterized by the encapsidation of viral genomic components by protein coats. Based on the architecture, organization and packaging of viral genomic components, viruses can be divided into three categories, namely monopartite, segmented and multipartite [3, 4]. Monopartite viruses have only one genomic component that is encapsidated in a protein coat. The genome of segmented viruses consists of two or more genomic components that are encapsidated together in a single virus particle. Multipartite viruses have several genomic components that are packaged into separate virus particles [3, 4]. Multipartitism is prevalent, especially in plant viruses, accounting for 30–40% of plant virus genera and families [3]. Studies in recent years have highlighted that the relative frequencies of the genomic components of multipartite viruses may vary significantly in virus life cycle [3, 5–7]. And more importantly, variation of the relative frequencies of viral genomic components may significantly impact the pathogenesis of viruses in their hosts [3, 4, 8–10].

Begomoviruses (family *Geminiviridae*) are a group of plant single-stranded DNA (ssDNA) viruses that are transmitted by whiteflies of the *Bemisia tabaci* complex [11, 12]. The genus *Begomovirus* is now the largest in the entire virosphere as recognized by the International Committee on Taxonomy of Viruses [11]. Moreover, in recent decades begomoviruses have exacted a heavy toll on the production of many solanaceous, cucurbitaceous, malvaceous and leguminous crops in warm and temperate regions around the globe [12]. Diseases caused by begomoviruses that are of particular significance include tomato yellow leaf curl, cotton leaf curl and cassava mosaic [12]. Begomoviruses can be either monopartite or bipartite depending on the number of genomic components [11]. The genome of bipartite begomoviruses consists of two circular ssDNA molecules of around 2.6 kb, referred to as DNA-A and DNA-B. Monopartite begomoviruses contain only one genomic component that resembles the DNA-A of bipartite begomoviruses [11]. Out of the 445 species proposed in the genus *Begomovirus*, 164 are bipartite, and the majority of them are distributed in the New World [13]. In recent years, an increase is witnessed in the incidence of crop diseases caused by bipartite begomoviruses in Asia such as squash leaf curl China virus (SLCCNV) and Sri Lankan cassava mosaic virus (SLCMV) [14–17]. Research efforts to disentangle the molecular biology of these viruses may help to combat them and thereby safeguard crop production.

Although the evolutionary origin of begomoviral DNA-A has not been unambiguously resolved, distinct origins of DNA-A and DNA-B have been proposed [18–20]. DNA-B was proposed to be originated as a satellite molecule that was later captured by monopartite begomoviruses or their progenitors [19]. Sequence analyses revealed that while always associated, DNA-A and DNA-B underwent distinct evolutionary histories, with DNA-B harboring more genetic variations resulted from mutation and recombination than DNA-A [19, 21]. DNA-A encodes proteins required for virus replication, gene expression regulation, encapsidation, whitefly transmission and host defense suppression, and DNA-B encodes two proteins that regulate intra- and intercellular virus movement in plants [11, 22]. Both DNA-A and DNA-B are required for virus infection in plants, with only a few exceptions [11, 23]. As for the movement of the two DNA components, it was found that DNA-A and DNA-B of abutilon mosaic virus, a bipartite begomovirus, spread within *N. benthamiana* plants independently during the early course of systemic infection, resulting in a stochastic distribution of DNA-A- and DNA-A/B-infected nuclei [24]. Nevertheless, while the importance of the association between DNA-A and DNA-B has been well established, the quantitative relationship between the two genomic components remain barely characterized. For example, how is the ratio between the two genomic components regulated during virus infection, and is this ratio affected by the process of virus transmission?

In this study, we examined the ratio between the two DNA components of bipartite begomoviruses during infection and transmission. First, we monitored the temporal dynamics of the quantities of SLCCNV DNA-A and DNA-B and their ratio in plants. Second, we examined the effects of B/A ratio in agrobacteria inoculum on the dynamics of the quantities of SLCCNV DNA-A and DNA-B and their ratio in plants. Third, we analyzed the impact of sap- and whitefly-mediated transmission on the quantities of SLCCNV DNA-A and DNA-B and their ratio in plants. Fourth, we analyzed the dynamics of the expression of genes encoded by SLCCNV DNA-A and DNA-B and their ratio in plants. Finally, we monitored the temporal dynamics of the quantities of DNA-A and DNA-B and the B/A ratio of another bipartite begomovirus SLCMV. Our results provide new insights into the biology of bipartite begomoviruses.

## Materials and methods

### Plants, viruses and whiteflies

Four species of plants were used, namely cotton (*Gossypium hirsutum* cv. Zhe-Mian 1793), zucchini (*Cucurbita pepo* cv. Faguodongkui), squash (*C. moschata* cv. Mibennangua), and tobacco (*Nicotiana benthamiana*). All plants were grown in insect-proof greenhouses under controlled temperature at 25 ± 3 ℃. Cotton plants were used for whitefly rearing, zucchini and squash plants were used for the experiments with SLCCNV, and tobacco plants were used for the experiments with SLCMV. For agrobacteria-mediated virus inoculation and virus transmission experiment, zucchini and squash plants at 1–2 true leaf stage were used, and tobacco plants at five true leaf stage were used. Two bipartite begomoviruses were used, namely SLCCNV (GenBank accession codes: MG525551 [DNA-A] and MG525552 [DNA-B]) and SLCMV (GenBank accession codes: OK571385 [DNA-A] and OK571386 [DNA-B]). Both SLCCNV isolate Guangxi2017 and SLCMV isolate Cambodia2015 were collected from field, and infectious clones were constructed as mentioned before [25, 26]. No viral satellite has been reported to be associated with the two bipartite begomoviruses in the field [14–17]. Agrobacteria containing infectious clones of DNA-A or DNA-B were first cultured separately until OD600 reached 1.5, and then pelleted and resuspended in resuspension buffer (10 mM MgCl_2_, 10 mM MES, 200 µM acetosyringone). Next, agrobacteria were mixed and introduced into all the true leaves and cotyledons of zucchini or squash plants, and the first true leaf at the bottom for tobacco plants. Agroinoculation was conducted with 1 ml syringe. For insects, a culture of Asia II 1 whiteflies of the *B. tabaci* complex (mt*COI* GenBank accession codes: DQ309077) was used. Whiteflies were originally collected from field and thereafter reared on cotton plants in insect-proof cages in climate chambers (26 ± 2 ℃, 60–80% relative humidity and 14/10 h light/dark cycles).

### Extraction of DNA and quantification of DNA-A and DNA-B in plants

DNA extraction was performed using Plant Genomic DNA Kit (Tiangen, China). Ensuring quantitative PCR(qPCR) analysis of viral DNA-A and DNA-B was performed using SYBR Green Premix Pro Taq HS qPCR Kit (Accurate Biology, China) and CFX96 Real-Time PCR Detection System (Bio-Rad, USA). The *actin* gene was used as an internal reference. Each gene was analyzed in duplicate technical repeats for each of the samples. The average threshold cycle (Ct) was calculated per sample. Primers are listed in Table 1.

**Table 1:** Primers used in this study

Primer	Sequence (5’-3’)	Application
SLCCNV-AV1-RTF	GAAGCGACCAGCCGATATTA	Quantification of SLCCNV DNA-A and *AV1*
SLCCNV-AV1-RTR	GGCACATCGGGACTTCTATA	
SLCCNV-BV1-RTF	ACGGGAACGAATAACAGGGT	Quantification of SLCCNV DNA-B and *BV1*
SLCCNV-BV1-RTR	CAATCGACACGACGCCATAA	
Zu-Actin-RTF	TTGCTGGTCGTGATCTGACT	Quantification of zucchini and squash *actin*
Zu-Actin-RTR	TGTCTCCAGTTCTTGCTCGT	
SLCMV-AV1-RTF	ACGCCAGGTCTGAGGCTGTA	Quantification of SLCMV DNA-A and *AV1*
SLCMV-AV1-RTR	GTTCAACAGGCCGTGGGACA	
SLCMV-BV1-RTF	TCGTTTCAAAGGCACTCGAC	Quantification of SLCMV DNA-B and *BV1*
SLCMV-BV1-RTR	TACGTCGCTGAGCCATACAT	
Nb-Actin-RTF	GCGAGTAAACCCGTAAGG	Quantification of tobacco *actin*
Nb-Actin-RTR	GCTCAGGCATAGTTCACC	

### Extraction of RNA and analysis of gene expression level

Total RNAs were extracted with TRIzol following user manual. cDNA was synthesized using Evo M-MLV RT Kit with gDNA Clean for qPCR (Accurate Biology, China). qPCR was performed as mentioned above with primers listed in Table 1.

### Analysis of temporal dynamics of the quantities of DNA-A and DNA-B and the expression of ***AV1*** and ***BV1*** in plants

To obtain virus-infected plants, equal quantity of agrobacteria containing infectious clones of DNA-A and DNA-B were mixed and then used for agroinoculation. Next, the first fully-expanded leaves of inoculated plants were collected at designated days post inoculation (dpi) and subjected to DNA extraction and the quantification of DNA-A and DNA-B. For SLCCNV-infected zucchini plants, at 12, 18, 24 and 30 dpi, RNA samples were similarly collected from the first fully-expanded leaves and subjected to RNA extraction and the quantification of *AV1*, *BV1*, and *actin* mRNA level. All plants were sampled only once.

### Analysis of the effects of B/A ratio in agrobacteria inoculum on quantities of DNA-A and DNA-B and their ratio in plants

To prepare agrobacteria inoculum with different B/A ratio (4, 1 and 1/4), agrobacteria containing infectious clones of DNA-A were mixed with different quantity of agrobacteria containing infectious clones of DNA-B. In these mixed solutions, the final OD600 value of agrobacteria containing infectious clones of DNA-A was kept constant. These agrobacteria solutions were then used for inoculation in zucchini plants. At 6, 18 and 30 dpi, the first fully-expanded leaves of the inoculated plants were collected and subjected to DNA extraction and DNA-A and DNA-B quantification.

### Analysis of the dynamics of quantities of DNA-A and DNA-B and their ratio in plants during sap- and whitefly-mediated virus transmission

SLCCNV-infected zucchini plants were obtained by agroinoculation using a mixture of equal quantity of agrobacteria containing infectious clones of DNA-A and DNA-B. SLCCNV-infected plants at 18 dpi were sampled and the remaining plants were used as the inoculum plants in sap- and whitefly-mediated virus transmission. For sap-mediated transmission, symptomatic leaves were collected, grounded in liquid nitrogen and dissolved in 0.01 M phosphate buffered saline (pH 7.4) (3:20, wt/vol). The resultant mixtures were filtered using medical gauze and then inoculated onto zucchini leaves by rubbing with carborundum powder (600 mesh). For whitefly-mediated transmission, whiteflies were collected and transferred to infected plants for a 4 days virus acquisition. Viruliferous whiteflies were then collected and released onto zucchini seedlings for a 4 days virus transmission using leaf-clip cages as mentioned before [26]. The number of whiteflies per test plants was ten. Post transmission, plants were all kept in climate chambers for 18 days, and then subjected to DNA extraction and DNA-A and DNA-B quantification.

### Statistical analysis

QPCR data of DNA-A and DNA-B quantity, and *AV1* and *BV1* expression were normalized to that of plant *actin* using 2^−∆Ct^ method. Next, B/A ratio or *BV1*/*AV1* were calculated for each sample. One-way analysis of variance (ANOVA) along with Fisher’s least significant difference (LSD) was used for the analysis of statistical significance. All data were presented as the mean ± standard errors of mean (mean ± SEM). Statistical analyses were conducted using SPSS Statistics 20.0 and EXCEL.

## Results

### Temporal dynamics of the quantities of SLCCNV DNA-A and DNA-B and their ratio in plants

**Fig. 1:**
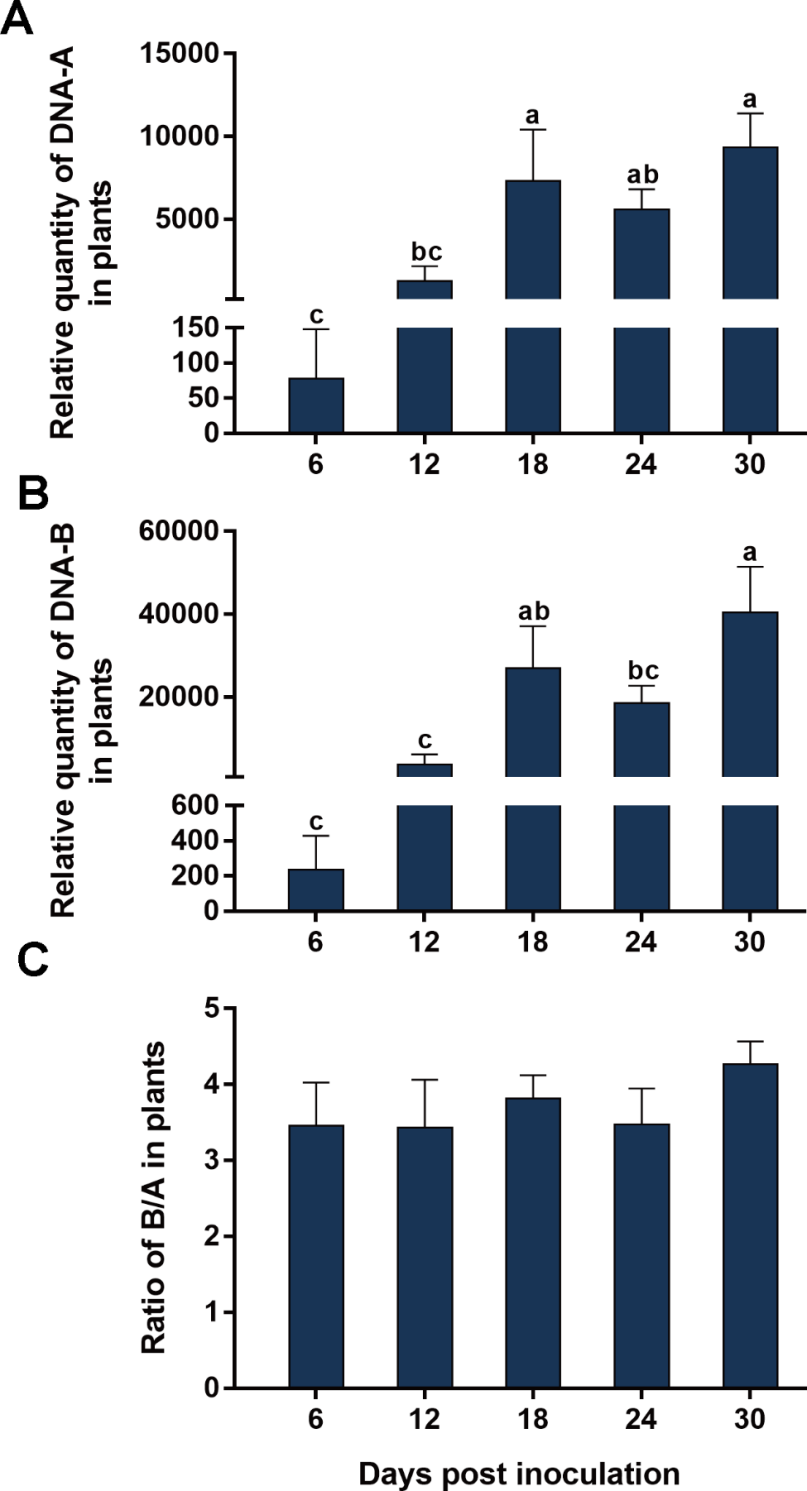
Temporal dynamics of the quantities of SLCCNV DNA-A and DNA-B and their ratio in zucchini plants. Zucchini plants were inoculated with a mixture of equal quantities of agrobacteria containing infectious clones of SLCCNV DNA-A and DNA-B, and then sampled at designated dpi for the quantification of DNA-A (A), DNA-B (B) and B/A ratio (C). Values are means ± SEM. The number of plants tested was 10 at 6 dpi, 6 at 12 dpi, 9 at 18 dpi, 8 at 24 and 30 dpi. Different letters above the columns indicate significant differences (one-way ANOVA, *P* < 0.05)

**Fig. 2:**
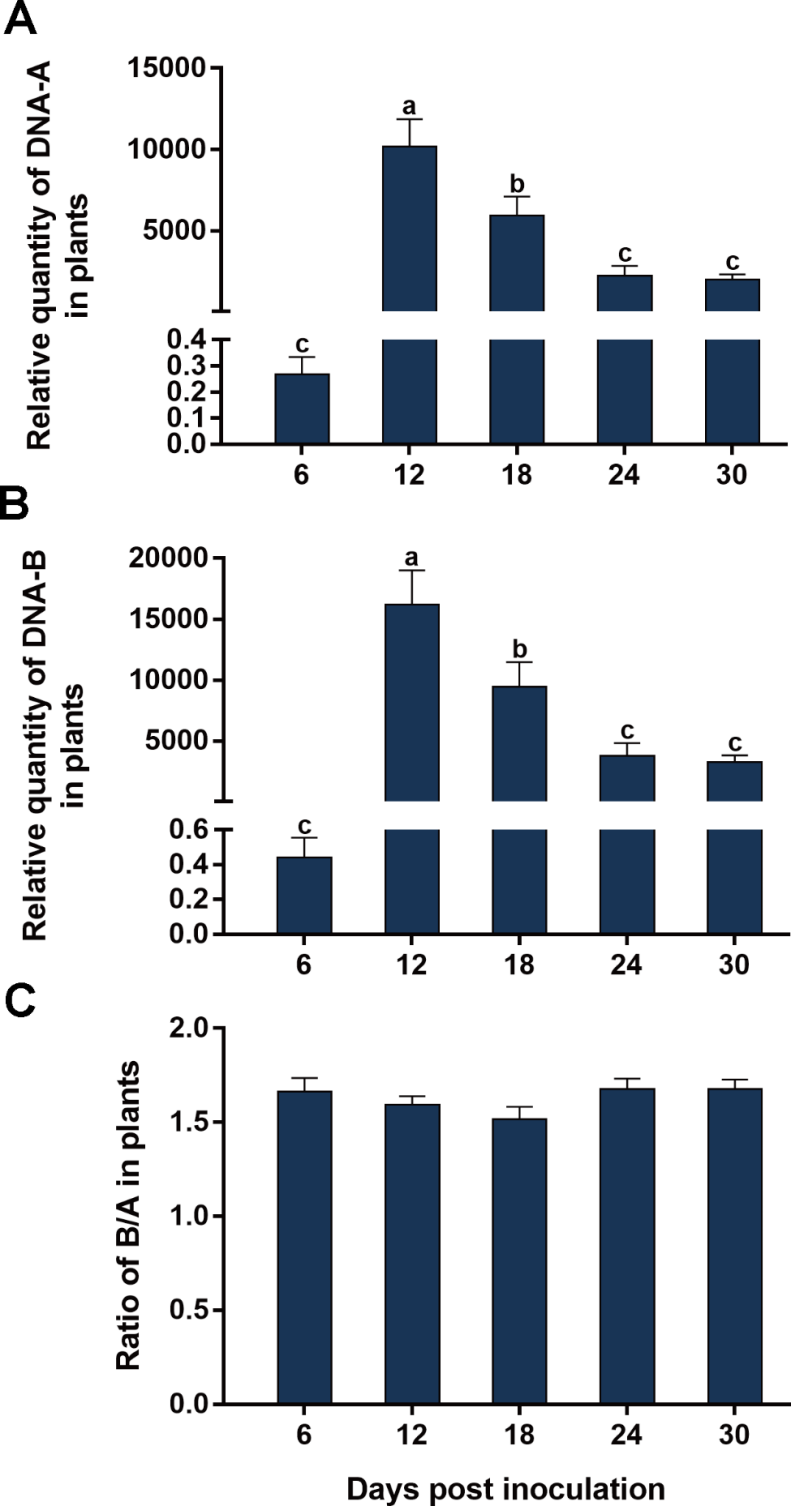
Temporal dynamics of the quantities of SLCCNV DNA-A and DNA-B and their ratio in squash plants. Squash plants were inoculated with a mixture of equal quantities of agrobacteria containing infectious clones of SLCCNV DNA-A and DNA-B; relative quantities of DNA-A and DNA-B and the B/A ratio (A, B and C). Values are means ± SEM. The number of plants tested was 10 at 6 dpi, 12 at 12, 18, 24 and 30 dpi. Different letters above the columns indicate significant differences (one-way ANOVA, *P* < 0.05)

We analyzed the temporal dynamics of the quantities of SLCCNV DNA-A and DNA-B and their ratio in zucchini and squash, two susceptible cucurbitaceous host plants of SLCCNV [26]. When zucchini plants were inoculated with a mixture of equal quantities of agrobacteria containing infectious clones of DNA-A and DNA-B, the quantities of DNA-A and DNA-B increased from 6 to 18 dpi and then changed only marginally from 18 to 30 dpi (Fig. 1A and B). When squash plants were inoculated, the relative quantities of DNA-A and DNA-B increased from 6 to 12 dpi, decreased from 12 to 24 dpi, and did not show significant changes from 24 to 30 dpi (Fig. 2A and B). Interestingly, in both zucchini and squash, the B/A ratios showed no significant changes from 6 to 30 dpi (Figs. 1 and 2 C, Table 2).

**Table 2:** Statistics of one-way ANOVA

Independent variable	Dependent variable	F	df	*P* *
Days post inoculation	SLCCNV DNA-A quantity in zucchini	4.561	4	**0.004**
	SLCCNV DNA-B quantity in zucchini	5.346	4	**0.002**
	SLCCNV B/A ratio in zucchini	0.558	4	0.694
	SLCCNV DNA-A quantity in squash	15.872	4	**< 0.001**
	SLCCNV DNA-B quantity in squash	13.685	4	**< 0.001**
	SLCCNV B/A ratio in squash	1.476	4	0.223
	SLCCNV *AV1* expression in zucchini	25.849	3	**< 0.001**
	SLCCNV *BV1* expression in zucchini	47.278	3	**< 0.001**
	SLCCNV *BV1*/*AV1* expression ratio in zucchini	4.724	3	**0.006**
	SLCMV DNA-A quantity in tobacco	13.775	3	**< 0.001**
	SLCMV DNA-B quantity in tobacco	6.260	3	**0.001**
	SLCMV B/A ratio in tobacco	0.485	3	0.694
DNA-B/DNA-A ratio in agrobacteria	SLCCNV DNA-A quantity in zucchini at 6 dpi	1.098	2	0.348
	SLCCNV DNA-B quantity in zucchini at 6 dpi	0.631	2	0.539
	SLCCNV B/A ratio in zucchini at 6 dpi	9.729	2	**< 0.001**
	SLCCNV DNA-A quantity in zucchini at 18 dpi	0.897	2	0.418
	SLCCNV DNA-B quantity in zucchini at 18 dpi	0.741	2	0.485
	SLCCNV B/A ratio in zucchini at 18 dpi	0.647	2	0.530
	SLCCNV DNA-A quantity in zucchini at 30 dpi	0.575	2	0.569
	SLCCNV DNA-B quantity in zucchini at 30 dpi	0.323	2	0.726
	SLCCNV B/A ratio in zucchini at 30 dpi	0.074	2	0.929
Methods of virus inoculation	SLCCNV DNA-A quantity in zucchini	75.585	2	**< 0.001**
	SLCCNV DNA-B quantity in zucchini	47.723	2	**< 0.001**
	SLCCNV B/A ratio in zucchini	1.716	2	0.194

* *P* values in bold indicate statistical significance

### Effects of B/A ratio in agrobacteria inoculum on quantities of SLCCNV DNA-A and DNA-B and their ratio in plants

**Fig. 3:**
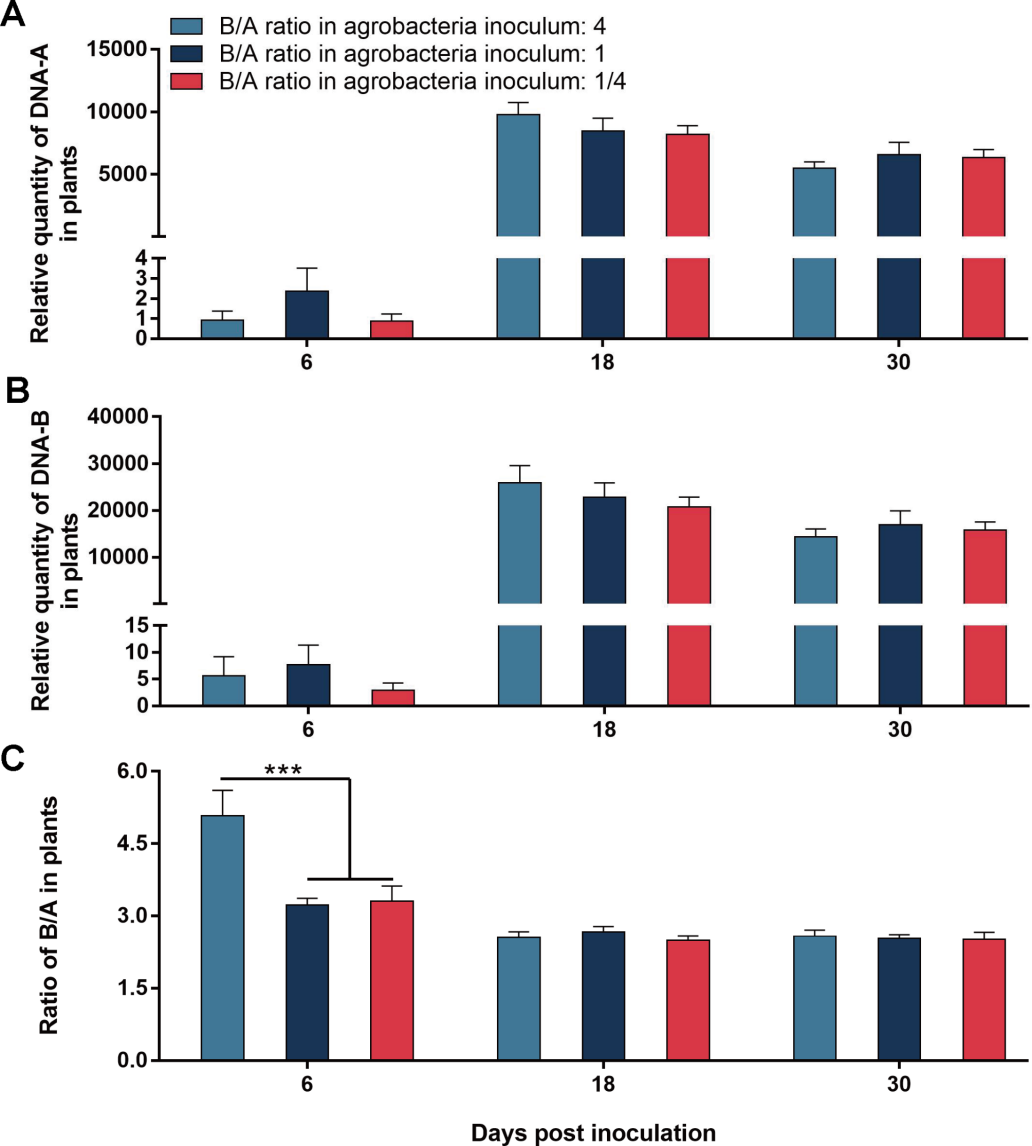
Effects of the B/A ratio in agrobacteria inoculum on the temporal dynamics of quantities of SLCCNV DNA-A and DNA-B and their ratio in zucchini plants. Agrobacteria containing infectious clones of SLCCNV DNA-A were mixed with different quantity of agrobacteria containing infectious clones of DNA-B. The final OD600 value of agrobacteria containing infectious clones of DNA-A was kept constant in these solutions; relative quantities of DNA-A and DNA-B, and the B/A ratio (A, B and C). Values are means ± SEM. The number of plants tested for the three treatments was 8, 12 and 10 at 6 dpi, 12, 11 and 11 at 18 dpi, 12, 12 and 11 at 30 dpi. *** indicates significant differences (one-way ANOVA, *P* < 0.001)

We prepared three agrobacterium inocula containing different ratios of B/A (4, 1 and 1/4), and kept the level of DNA-A constant. For both DNA-A and DNA-B, B/A ratio in agrobacterium inocula did not significantly affected their quantities at 6, 18 and 30 dpi, respectively (Fig. 3A, B; Table 2). A significantly higher B/A ratio was found in plants inoculated with the inoculum with the highest ratio of B/A at 6 dpi. However, the difference disappeared at 18 and 30 dpi (Fig. 3C; Table 2).

### Dynamics of the quantities of SLCCNV DNA-A and DNA-B and their ratio in plants during transmission

**Fig. 4:**
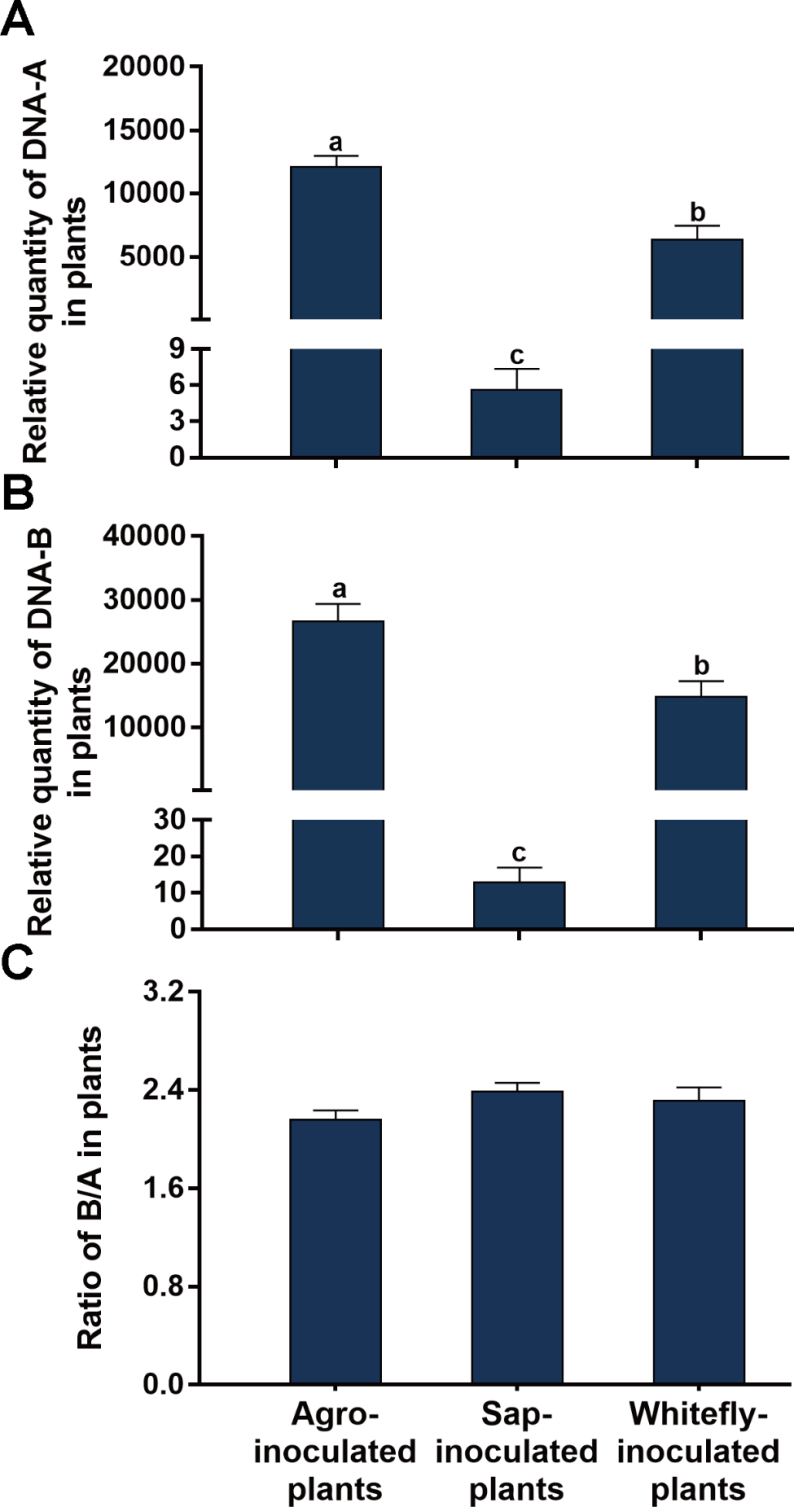
Quantities of SLCCNV DNA-A and DNA-B and their ratio in zucchini plants that were inoculated by agrobacteria, sap and whitefly. Zucchini plants were inoculated with a mixture of equal quantities of agrobacteria containing infectious clones of SLCCNV DNA-A and DNA-B. At 18 dpi, some plants were sampled and the others were used as the source of inoculum for sap- and whitefly-mediated virus transmission. At 18 days post transmission, sap- and whitefly-inoculated plants were sampled; relative quantities of DNA-A and DNA-B and the B/A ratio (A, B and C). Values are means ± SEM. The number of plants tested was 15 for agrobacteria and sap treatments, 10 for whitefly treatment. Different letters above the columns indicate significant differences (one-way ANOVA, *P* < 0.05)

We compared the quantities of DNA-A and DNA-B among plants of three treatments of virus inoculation: agrobacteria-inoculation, sap-mediated transmission and whitefly-mediated transmission. The methods of virus inoculation significantly affected the quantities of DNA-A and DNA-B, but not the B/A ratio (Fig. 4; Table 2). Specifically, the highest quantity of DNA-A and DNA-B was found in agrobacteria-inoculated plants, followed by the whitefly-inoculated plants, and then the sap-inoculated plants (Fig. 4A and B).

### Temporal dynamics of the expression of SLCCNV ***AV1*** and ***BV1*** and their ratio in plants

**Fig. 5:**
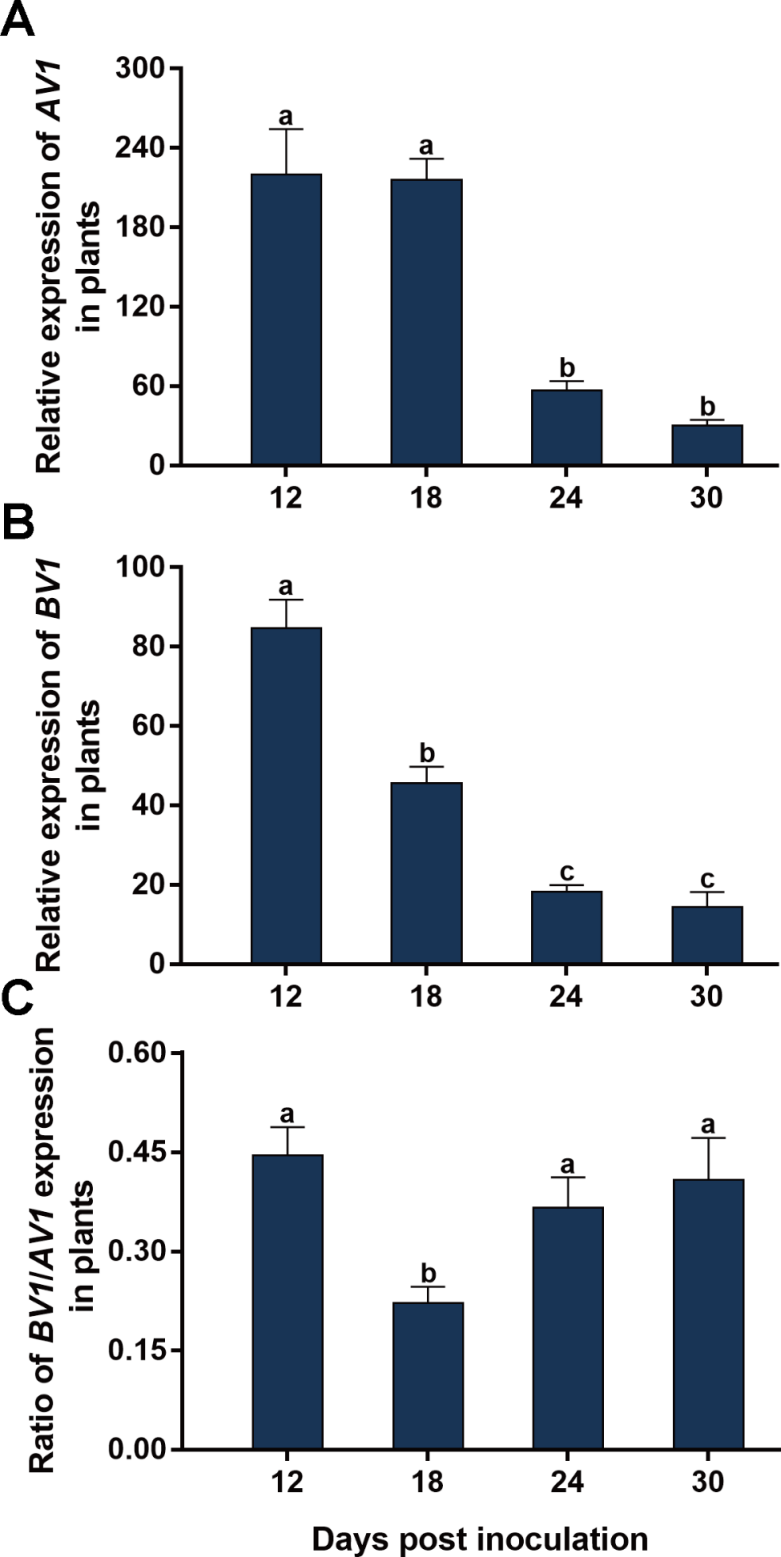
Temporal dynamics of expression of SLCCNV ***AV1*and*BV1*and their ratio in zucchini plants.** Zucchini plants were inoculated with a mixture of equal quantities of agrobacteria containing infectious clones of SLCCNV DNA-A and DNA-B; relative expressions of *AV1* and *BV1* and *BV1*/*AV1* expression ratio (A, B and C). Values are means ± SEM. The number of plants tested was 12 at 12, 18 and 24 dpi, and 11 at 30 dpi. Different letters above the columns indicate significant differences (one-way ANOVA, *P* < 0.05)

At 6 dpi, very low level of *AV1* and *BV1* expression was detected and thus only the data at 12, 18, 24 and 30 dpi were analyzed and presented. The relative expression of *AV1* was similar at 12 and 18 dpi, decreased significantly from 18 to 24 dpi, and did not show significant changes from 24 to 30 dpi (Fig. 5A). The relative expression of *BV1* decreased from 12 to 24 dpi and then did not show significant change from 24 to 30 dpi (Fig. 5B). The ratio of *BV1* expression to that of *AV1* decreased significantly from 12 to 18 dpi, increased from 18 to 24 dpi and then did not show significant change from 24 to 30 dpi (Fig. 5C). These data indicate that dpi significantly affected the expression of *AV1* and *BV1* and their ratio (Fig. 5; Table 2).

### Temporal dynamics of the quantities of SLCMV DNA-A and DNA-B and their ratio in plants

**Fig. 6:**
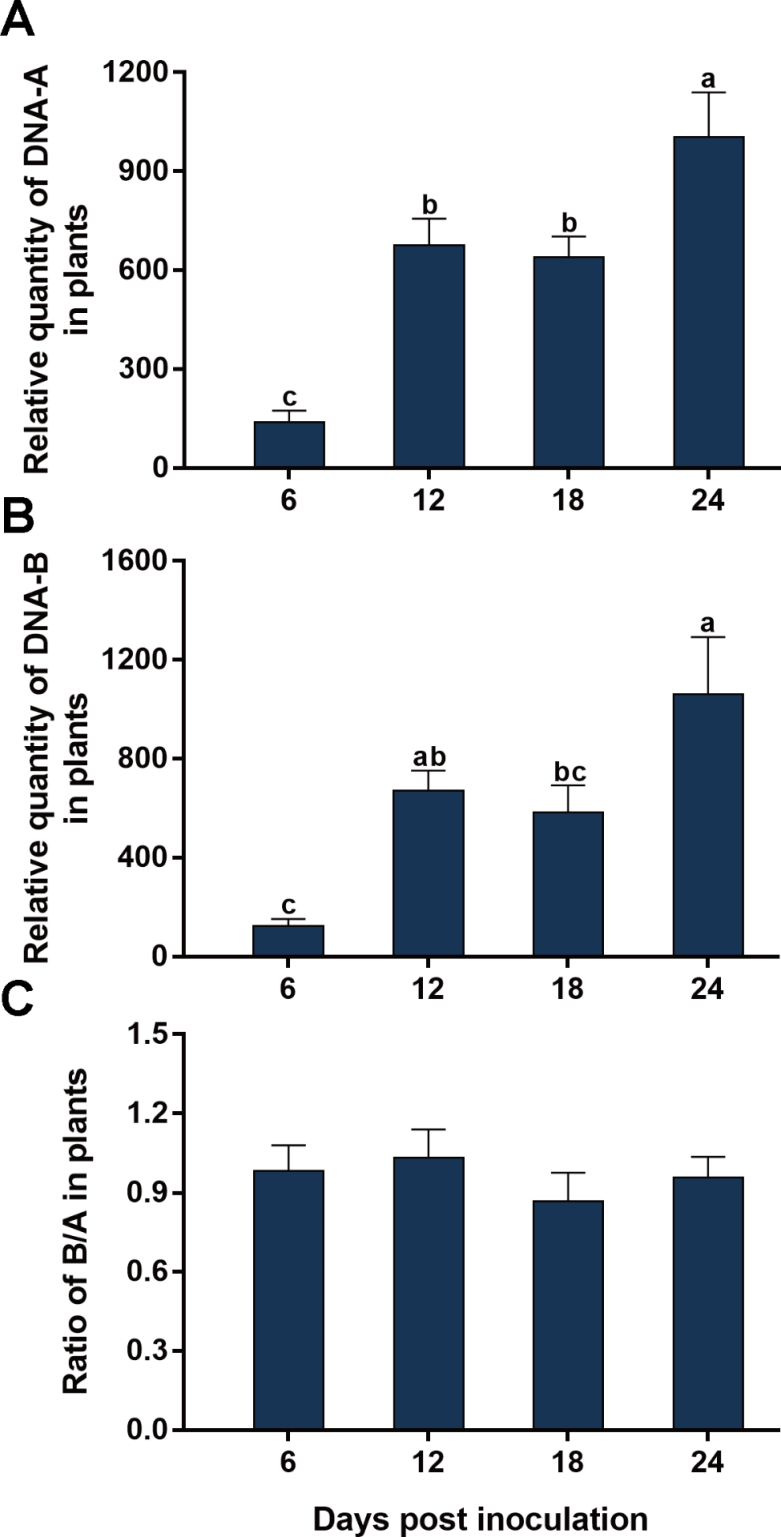
Temporal dynamics of quantities of SLCMV DNA-A and DNA-B and their ratio in tobacco plants. Tobacco plants were inoculated with a mixture of equal quantities of agrobacteria containing infectious clones of SLCMV DNA-A and DNA-B, and then sampled at designated dpi for the quantification of DNA-A (A), DNA-B (B) and B/A ratio (C). Values are means ± SEM. The number of plants tested was 14 at 6, 12 and 18 dpi, and 19 at 24 dpi. Different letters above the columns indicate significant differences (one-way ANOVA, *P* < 0.05)

When tobacco plants were inoculated with a mixture of equal quantities of agrobacteria containing infectious clones of SLCMV DNA-A and DNA-B, dpi significantly affected the quantities of DNA-A and DNA-B, but not their ratio (Fig. 6; Table 2). Specifically, DNA-A quantity increased from 6 to 12 dpi, and did not change significantly from 12 to 18 dpi, and then increased significantly from 18 to 24 dpi (Fig. 6A). DNA-B quantity increased from 6 to 12 dpi, and did not show significant change from 12 to 18 dpi, and then increased from 18 to 24 dpi (Fig. 6B).

## Discussion

In this study, we characterized the ratio between the two genomic components of bipartite begomoviruses. We first showed that when two plant species were inoculated with a mixture of equal quantities of agrobacteria inoculum containing infectious clones of SLCCNV DNA-A and DNA-B, the quantities of DNA-A and DNA-B varied significantly with time; However in each of the two plant species the B/A ratio remained constant during SLCCNV infection (Figs. 1 and 2; Table 2). We then inoculated zucchini plants with agrobacteria inocula of different B/A ratios of SLCCNV and observed the B/A ratio during infection. We demonstrated that while the variation of B/A ratio in agrobacteria inoculum may affect the B/A ratio in plants in the initial phase of infection, the effects subsequently disappeared (Fig. 3; Table 2). We further showed while the quantities of DNA-A and DNA-B in the plants infected by agrobacteria–inoculum, sap-mediated inoculation, and whitefly-mediated transmission differed significantly, the B/A ratios did not differ among plants infected with the three modes of virus inoculation (Fig. 4; Table 2). We next found that the expression of the gene *BV1* encoded by DNA-B and the gene *AV1* encoded by DNA-A and their ratio varied significantly during infection (Fig. 5; Table 2). Finally, we tested another bipartite begomovirus SLCMV, and similarly found that while the quantities of DNA-A and DNA-B varied significantly during infection, the B/A ratio remained constant (Fig. 6; Table 2).

For bipartite begomoviruses, DNA-A and DNA-B share no sequence similarity, except for a highly conserved common region of ∼200 nt that contains the viral origin of replication [18, 19]. During replication, Rep proteins encoded by DNA-A bind specifically to the replication origin of viral DNAs and initiate rolling circle replication [27, 28]. In this study, we found that B/A ratio of bipartite begomoviruses in plants is constant under various scenarios, indicating that the accumulation of DNA-A and DNA-B is tightly associated. The tightly-associated accumulation of the two genomic components may be attributable to the activity of Rep proteins, which are responsible for the replication of both DNA-A and DNA-B. Notably, B/A ratio of SLCCNV in two plant species is always higher than 1, suggesting that more DNA-Bs were constantly generated than DNA-As. For another bipartite begomovirus SLCMV, the B/A ratio in plant is ∼1. These results suggest that at least for some bipartite begomoviruses, Rep proteins do not replicate DNA-A and DNA-B randomly, as random replication of DNA-A and DNA-B is likely to result in similar quantities of the two genomic components. As the interaction between Rep proteins and common regions dictates viral DNA replication, we propose that higher accumulation of DNA-B than DNA-A may be due to the different affinity of the two common regions to Rep proteins. Additionally, the fact that DNA-A-encoded Rep proteins replicate DNA-B with efficiencies equal to or higher than that of DNA-A, indicates that the two genomic components of bipartite begomoviruses have adapted well to each other. Considering the disparate evolutionary origins of the two genomic components [18–20], it would be of interest to decipher how they have adapted to each other in the long-term association.

As the genomic components of viruses serve as the template for viral gene expression, changes in the copy number of certain viral genomic components may directly impact viral gene expression. In theory, multipartitism may promote the regulation of the expression of genes encoded by different genomic components [3, 4]. By changing the ratio of genomic components, namely the relative copy number of viral genes, the genes encoded by over-represented genomic components may be expressed preferentially to facilitate adaptation [3, 29]. For example, changes in the frequency of a given genomic component positively and linearly affected the expression of the corresponding genes in FBNSV, a multipartite nanovirus [29]. In our study, B/A ratio remained constant and between 3.42 and 4.26 in zucchini plants, indicating higher accumulation of DNA-B than that DNA-A. On the contrary, characterization of mRNA level shows higher expression of DNA-A-encoded *AV1* than DNA-B-encoded *BV1* at all the time points analyzed. While more investigations are required to explore the mechanisms underlying the preferred expression of *AV1*, we propose that the activity of promoters of the two viral genes may play a role as they directly dictate viral gene expression. More importantly, while the B/A ratio at DNA level remained constant, *BV1*/*AV1* expression ratio changed significantly during infection. Together, it seems that unlike FBNSV, B/A ratio of bipartite begomoviruses in plants may play a minor role, if any, in modulating the expression of genes encoded by the two genomic components. The reason of divergence may be that while each of the eight genomic components of FBNSV encodes only one gene, each genomic component of bipartite begomoviruses encodes more than one gene [8, 11]. Under this scenario, an exquisite and finely-tuned gene expression regulation system, instead of the simple B/A ratio in plants, is required for the precise control of viral gene expression in bipartite begomoviruses. Additionally, these findings suggest that cautions should be taken when applying knowledge obtained from multipartite viruses to studies of bipartite begomoviruses.

Taken together, here we have found that the B/A ratio of bipartite begomoviruses in plants remains constant during infection. Our results show that bipartite begomoviruses exert control on the B/A ratio in plants as the variation of B/A ratio in agrobacteria inoculum, sap transmission and whitefly-mediated virus transmission can barely affect the B/A ratio in plants as the infection progresses. Our findings uncovered a new feature of the genomes of bipartite begomoviruses, namely a constant quantitative relationship between the two genomic components during infection and transmission. This feature may play a role in the biology of bipartite begomoviruses, and should be subjected to further investigation for its contribution to viral pathogenesis.

### Electronic supplementary material

Below is the link to the electronic supplementary material.

Supplementary Material 1

Supplementary Material 2

Supplementary Material 3

Supplementary Material 4

Supplementary Material 5

Supplementary Material 6

## Data Availability

The datasets supporting the conclusions of this article are included within the article and its additional files.
